# Incremental Shuttle Walking Test Distance Is Reduced in Patients With Pulmonary Hypertension in World Health Organisation Functional Class I

**DOI:** 10.3389/fmed.2018.00172

**Published:** 2018-06-21

**Authors:** Catherine G. Billings, Robert Lewis, Iain J. Armstrong, Judith A. Hurdman, Ian A. Smith, Matthew Austin, Charlie A. Elliot, Athanasios Charalampopoulos, Ian Sabroe, Allan Lawrie, A. A. Roger Thompson, Robin Condliffe, David G. Kiely

**Affiliations:** ^1^Sheffield Pulmonary Vascular Disease Unit, Sheffield Teaching Hospitals NHS Foundation Trust, Royal Hallamshire Hospital, Sheffield, United Kingdom; ^2^Department of Infection, Immunity and Cardiovascular Disease, University of Sheffield, Medical School, Sheffield, United Kingdom; ^3^Insigneo Institute for in Silico Medicine, University of Sheffield, Sheffield, United Kingdom

**Keywords:** incremental shuttle walk test, pulmonary hypertension, WHO functional class, screening, early diagnosis, hemodynamics, exercise testing

## Abstract

**Background:** There is increasing interest in screening for and diagnosing pulmonary hypertension earlier in the course of disease. However, there is limited data on cardiopulmonary abnormalities in patients with pulmonary hypertension newly diagnosed in World Health Organization Function Class (WHO FC) I.

**Methods:** Data were retrieved from the ASPIRE registry (Assessing the Spectrum of Pulmonary hypertension Identified at a REferral center) for consecutive treatment naïve patients diagnosed with pulmonary hypertension by cardiac catheterization between 2001 and 2010 who underwent incremental shuttle walk exercise testing.

**Results:** Eight hundred and ninety-five patients were diagnosed with Group 1-5 pulmonary hypertension. Despite the absence of symptoms, patients in WHO FC I (*n* = 9) had a significant reduction in exercise capacity (Incremental shuttle walk distance percent predicted (ISWD%pred) 65 ± 13%, Z score −1.77 ± 1.05), and modest pulmonary hypertension with a median (interquartile range) pulmonary artery pressure 31(20) mmHg and pulmonary vascular resistance 2.1(8.2) Wood Units, despite a normal diffusion of carbon monoxide adjusted for age and sex (DLco)%pred 99 ± 40%. Compared to patients in WHO FC I, patients in WHO FC II (*n* = 162) had a lower ISWD%pred 43 ± 22 and lower DLco%pred 65 ± 21%.

**Conclusion:** Our results demonstrate that patients with newly diagnosed pulmonary hypertension with no or minimal symptomatic limitation have a significant reduction of exercise capacity.

## Introduction

Despite advances in treatment, pulmonary hypertension (PH) remains a progressive life-limiting disease (1). Studies have suggested that earlier intervention results in better outcomes (2, 3). However, patients are usually diagnosed when the disease is advanced (4, 5) and consequently there is interest in developing strategies to enable earlier diagnosis (6). To diagnose pulmonary hypertension prior to the development of significant disease, it is recommended that people at increased risk of developing pulmonary hypertension such as patients with systemic sclerosis should be screened (7). These international guidelines emphasize the importance of echocardiography in the screening process. In systemic sclerosis where the prevalence of pulmonary arterial hypertension is particularly high, investigators have also recommended the use of diffusion capacity of the lung for carbon monoxide percent predicted (DLco%pred) which is frequently reduced in patients with systemic sclerosis and pulmonary arterial hypertension (8–10). More recently a cross-sectional, international study looked at a large number of candidate biomarkers in systemic sclerosis to construct a model to aid decisions to proceed to cardiac catheterisation (the DETECT study) (11).

At rest the pulmonary circulation has large microcirculatory reserves. These are recruited during exercise, increasing the capillary surface area available for gas exchange and maintaining a low pulmonary artery pressure despite increased flow (12). Any reduction in pulmonary vasculature reserves may therefore be first detected during exercise. Although exercise testing using the 6-min walk test was included as a candidate marker in the DETECT study, and may have been expected to contribute to an early diagnostic model, it had no utility in the model constructed to diagnose pulmonary arterial hypertension. This may reflect the inability of the 6-min walk test to identify the presence of early pulmonary vascular disease given its ceiling effect where in mild disease 6 min walking test distance no longer reflects maximal oxygen aerobic capacity (13–15) or disease severity (16) or be due to an inability of patients with systemic sclerosis to exercise as a consequence of musculoskeletal disease. In addition, a systematic review of studies looking at the correlation of the New York Heart Association (NYHA) Classification and the 6-min walk distance (6MWD) in patients with heart failure without musculoskeletal disease (17) also found no significant difference between asymptomatic/mildly symptomatic patients (NYHA I and II).

The incremental shuttle walking test (ISWT) has no ceiling effect (18) and correlates better with peak exercise capacity than the 6-min walking test (19). We have hypothesized that the incremental shuttle walking test will be reduced in patients with pulmonary hypertension in World Health Organization functional class I (WHO FC I) when the patients have either no or minor symptoms of breathlessness.

## Methods

Data were retrieved from the ASPIRE registry (Assessing the Spectrum of Pulmonary hypertension Identified at a REferral center) for consecutive, treatment naïve patients diagnosed with pulmonary hypertension between 2001 and 2010 (4). The systematic assessment of the patients has previously described in detail (4). Patients included in this retrospective study were diagnosed as Group 1–5 PH and were required to have mean pulmonary artery pressure at right heart catheterisation of at least 25 mmHg and had a baseline ISWT within 3 months of cardiac catheterization.

### Incremental shuttle walk test

The ISWT was performed according to the method of Singh et al. (20). Patients were asked to walk as far as possible around the 10 m course keeping in time to an audio signal until they were too breathless or could no longer keep up with the speed. The initial walking speed was 0.50 m/s and this increased incrementally every minute to a maximum of 2.37 m/s. Breathlessness was measured at rest and at the end of the test using the modified Borg scale. Heart rate was measured throughout the test. ISWT distance (ISWD) percent predicted (ISWD%pred) and z score were calculated for each patient based on sex, age and BMI using the equation derived by Probst et al. (21). No supplemental oxygen was used during testing. If patients could not walk or could not walk without oxygen the distance was recorded as 0 m.

### Lung function tests

Lung function tests were performed in accordance to the European Respiratory Society guidelines (22–25). Predicted DLco (DLco%pred) and z score were calculated for each patient using the Global Lung Function Initiative (GLI) reference equations (26).

### Statistical analysis

Statistical analysis was performed using IBM SPSS Statistics v19 (SPSS, Chicago, IL, USA). Data is presented as mean ± SD for parametric data and median (interquartile range) for nonparametric data. Categorical variables were presented as %. To evaluate our hypothesis that the incremental shuttle walking test will be reduced in WHO FC I, the main outcome variable was ISWD%pred. DLco%pred was a secondary outcome to be used in comparison to the ISWD. Pearson's correlation test was used to assess correlations between these parameters and WHO FC and hemodynamic parameters. The student *t*-test and Mann Whitney U-test and Kruskal-Wallis test were used to compare groups. Event (death or transplantation) free survival from date of diagnosis was estimated using the Kaplan–Meier method with comparison between groups performed by the log-rank test. Cox proportional hazards regression analysis was used to assess the effect of ISWD%pred, age, sex, BMI, mPAP and DLco on survival time. As left to right shunt is known to result in high DLco%pred (27), separate analyses were also performed omitting patients with congenital heart disease. Post-hoc analyses were also performed on the group of patients in WHO FC I and II with pulmonary hypertension related to systemic sclerosis who had undergone screening for PH. A *p*-value of < 0.05 was deemed statistically significant. Ethical approval was granted by the North Sheffield Research Ethics Committee (Reference No. 06/Q2308/8).

## Results

**Figure 1:**
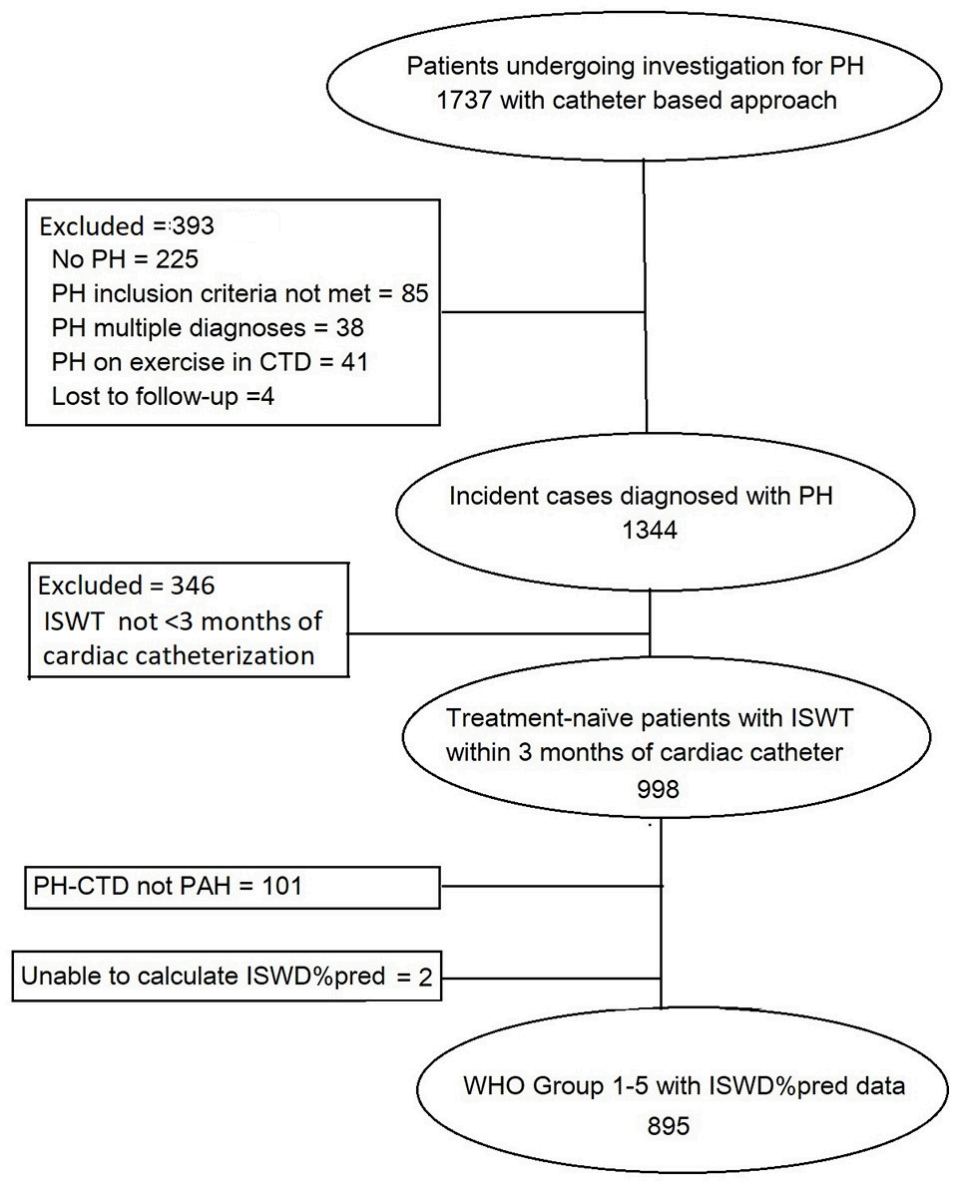
Patient flow diagram.

During the duration of the study 895 patients were diagnosed with pulmonary hypertension and met the entry criteria (Figure 1). At diagnosis 9 patients (1%) were in WHO functional class 1, and 162 (18%), 592 (66%), and 132 (15%) in WHO Functional Class II, III, and IV, respectively. Of those in WHO FC I, 3 had systemic sclerosis (one of whom had a PAWP > 15 mmHg), 3 had congenital heart disease with left to right shunts, 1 left heart disease, 1 chronic thromboembolic pulmonary hypertension and 1 hereditary pulmonary arterial hypertension.

### Demographics and pulmonary hemodynamics

Patients' characteristics are shown in Table 1. Patients in WHO FC I were not significantly different to patients in the other WHO FC's with respect to age, sex distribution or smoking history. No patients in WHO FC I were on oxygen therapy compared with 6% in WHO FC II. Patients in WHO FC I had modest pulmonary hypertension: mPAP median(Interquartile range) 31(20) mmHg, preserved cardiac index (CI): mean ± standard deviation 3.4 ± 1.8 l/min/m^2^ with no significant difference compared to WHO FC II.

**Table 1:** Patient characteristics and hemodynamic parameters.

	**WHO functional class**			
	**I**	**II**	**III**	**IV**
n	9	162	592	132
Sex male/female %	44/56	46/54	38/62	37/63
Age years	51 ± 13	56 ± 16	61 ± 16^*^	64 ± 16
BMI kg/m2	28.2 ± 4.7	28.0 ± 6.1	27.7 ± 6.2	27.1 ± 6.6
Smoking % Never/Ex/Current	43/57/0	48/44/8	38/51/12	36/57/7
Pack years	10 ± 16	12 ± 19	17 ± 20	25 ± 30^†^
Oxygen therapy % None/LTOT/Other	100/0/0	89/6/5	62/32/7	26/68/6
**HEMODYNAMICS**				
mRAP mmHg	8(8)	8(7)	9(8)	12(8)^†^
mPAP mmHg	31(20)	37(16)	45(17)	47(12)
sPAP mmHg	51(38)	58(25)	74(29)	75(24)
Wedge mmHg	16 ± 9	13 ± 6	12 ± 6	11 ± 5
CI L/min/m^2^	3.4 ± 1.8	3.1 ± 0.8	2.7 ± 0.8^*^	2.1 ± 0.6^†^
PVR Wood Unit	2.1(8.2)	3.6(3.8)	7.0(6.9)^*^	11.5(6.8)^†^
SmvO2 %	67 ± 6	67 ± 7	63 ± 9^*^	58 ± 9^†^
**ISWT**				
ISWD m	450(150)	280(258)^▴^	120(140)^*^	10(58)^†^
ISWD%pred	65(20)	42(28)^▴^	20(21)^*^	3(9) ^†^
ISWD Z score	−1.77 ± 1.05	−2.36 ± 1.36^▴^	−3.11 ± 1.14^*^	−3.48 ± 1.10^†^
Starting SaO2 %	96.7 ± 2.1	93.8 ± 4.2^▴^	91.0 ± 6.3^*^	85.0^†^
Lowest SaO2 %	84.2 ± 10.5	83.4 ± 11.0	82.6 ± 11.2	79.1 ± 11.6^†^
Starting HR bpm	85. ± 14	82 ± 17	83 ± 17	85 ± 18
Highest HR bpm	144 ± 37	125 ± 30	114 ± 23^*^	110 ± 23
Resting SBP mmHg	125 ± 25	131 ± 22	127 ± 20^*^	117 ± 19^†^
Highest SBP mmHg	159 ± 11	158 ± 25	145 ± 27^*^	128 ± 24^†^
**Borg dyspnea score**				
Pre-test	0.6 ± 0.7	0.7 ± 1.0	1.1 ± 1.3^*^	1.8 ± 1.4^†^
Post-test	3.8 ± 1.6	4.2 ± 1.9	4.3 ± 1.9	4.7 ± 2.0^†^
**LUNG FUNCTION**				
FEV1%pred	97 ± 14	75 ± 23^▴^	74 ± 21	67 ± 23^†^
FVC%pred	103 ± 17	87 ± 23	87 ± 23	81 ± 27
DLco mmol/min/kPa	7.6 ± 3.2	5.6 ± 2.2^▴^	4.3 ± 2.0^*^	2.9 ± 1.7^†^
DLco%pred	99 ± 40	72 ± 24^▴^	59 ± 22^*^	41 ± 21^†^
DLco Z score	−0.57 ± 3.43	−2.22 ± 1.96^▴^	−3.45 ± 2.37^*^	−5.69 ± 3.10^†^

Presented as mean ± SD for parametric data and median(interquartile range) for nonparametric data. Categorical variables were presented as %. ISWT, incremental shuttle walk test; WHO FC, World Health Organisation functional class; BMI, body mass index; mRAP, mean right atrial pressure; mPAP, mean pulmonary arterial pressure; sPAP, systolic pulmonary arterial pressure; CI, cardiac index; PVR, pulmonary vascular resistance; SmvO2, mixed venous oxygen saturation; ISWD, incremental shuttle walk test distance; %pred: percent predicted; SaO2, oxygen saturation; HR, heart rate; SBP, systolic blood pressure; FEV1, forced expiratory volume in 1 second; FVC, forced vital capacity; DLco, diffusion factor across the lung for carbon monoxide; z score, standardized score; ^▴^p < 0.05 WHO FC I vs. WHO FC II;

* p < 0.05 WHO FC II vs. WHO FC III;

† *p < 0.05 WHO FC III vs. WHO FC IV*.

### Incremental shuttle walking test and lung function testing

Incremental shuttle walking distances (absolute, %pred and standardized (z) score) were all significantly higher in WHO FC I compared to WHO FC II. In addition, resting oxygen saturation pre-test was significantly higher in WHO FC I compared to WHO FC II. There was no significant difference in Borg dyspnea score between these 2 groups either pre- or post-ISWT and no significant difference in highest heart rate or highest systolic blood pressure measured. ISWD%pred correlated significantly (*p* all < 0.05) with WHO FC, mRAP, mPAP, sPAP, CI, PVR, and SmVO_2_ (Table 2).

**Table 2:** Correlation of ISWD%pred with WHO FC, hemodynamics, dyspnea score and DLco%pred.

	***N***	***r***	**95% CI**		***p***
WHO FC	895	−0.576	−0.598	−0.553	< 0.001
mRAP	778	−0.238	−0.301	−0.175	< 0.001
sPAP	762	−0.145	−0.214	−0.075	< 0.001
mPAP	777	−0.171	−0.239	−0.103	< 0.001
PVR	755	−0.263	−0.327	−0.195	< 0.001
CI	754	0.271	0.139	0.336	< 0.001
SmvO2	762	0.284	0.217	0.348	< 0.001
Wedge	724	0.014	−0.059	0.087	0.697
Borg Pre	631	−0.366	−0.432	−0.297	< 0.001
Borg Post	617	0.032	0.111	−0.047	0.433
DLco%pred	815	0.371	0.311	0.413	< 0.001

*ISWD%pred, Incremental Shuttle Walk Test distance percent predicted; WHO FC, World Health Organization functional class; mRAP, mean right atrial pressure; sPAP, systolic pulmonary arterial pressure; mPAP, mean pulmonary arterial pressure; PVR, pulmonary vascular resistance; CI, cardiac index; SmvO2, mixed venous oxygen saturation; ISWD, Incremental Shuttle Walk Test distance; %pred, percent predicted; DLco%pred, diffusion factor across the lung for carbon monoxide percent predicted*.

There were also significant differences in lung function between WHO FC I and II. DLco, DLco%pred, DLco z score and FVC were all significantly higher in WHO FC I. DLco%pred correlated with WHO FC, CI, PVR, and SmVO_2_ but not mRAP, mPAP, or sPAP. There was a stronger correlation of WHO FC with ISWD%pred compared with correlation to DLco%pred, −0.576 and −0.382, respectively.

**Figure 2:**
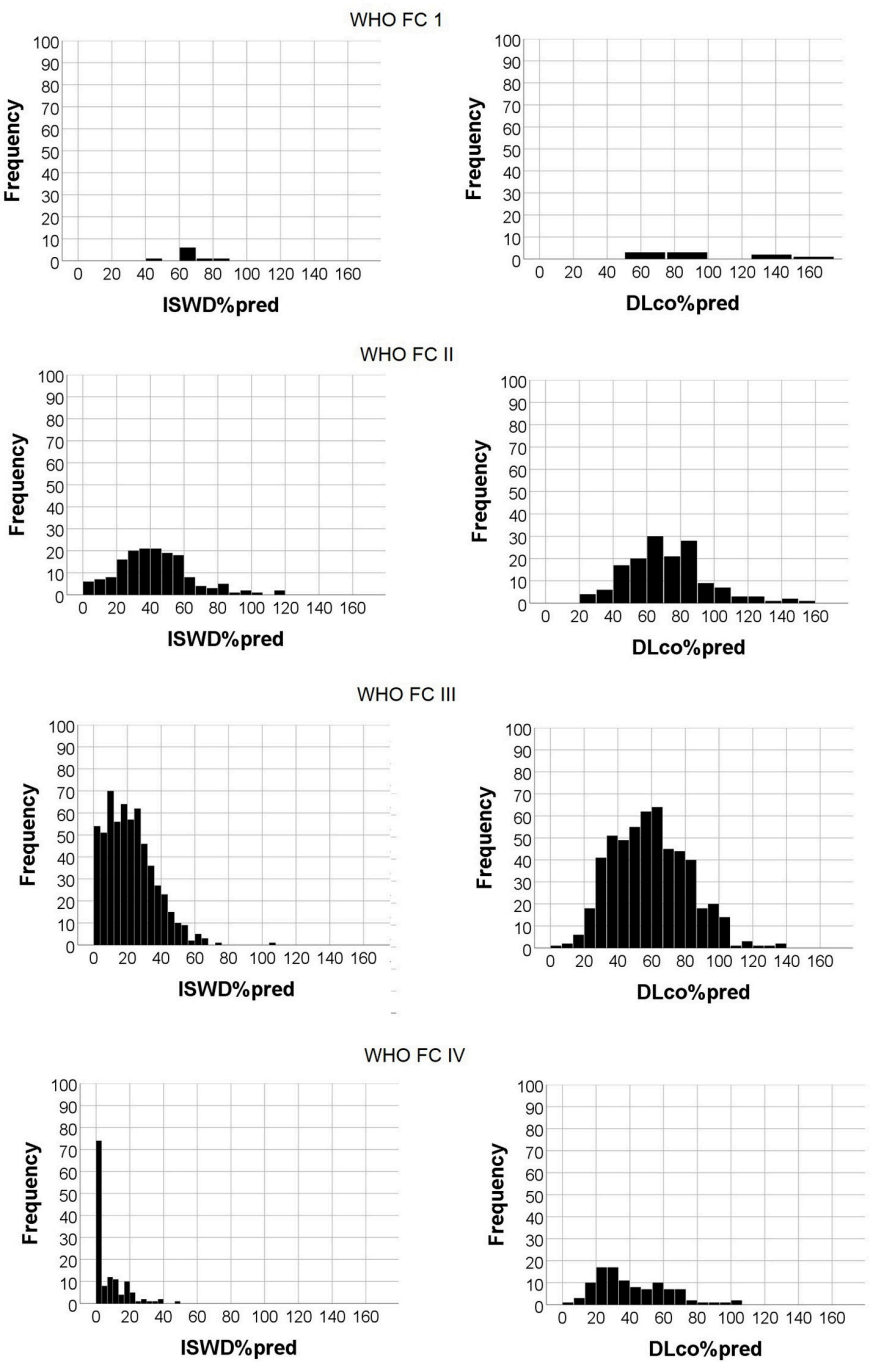
Histogram of Incremental shuttle walk distance percent predicted (ISWD%pred) and Diffusing capacity across the lung for carbon monoxide percent predicted (DLco%pred) frequencies by World Health Organization functional class.

**Figure 3:**
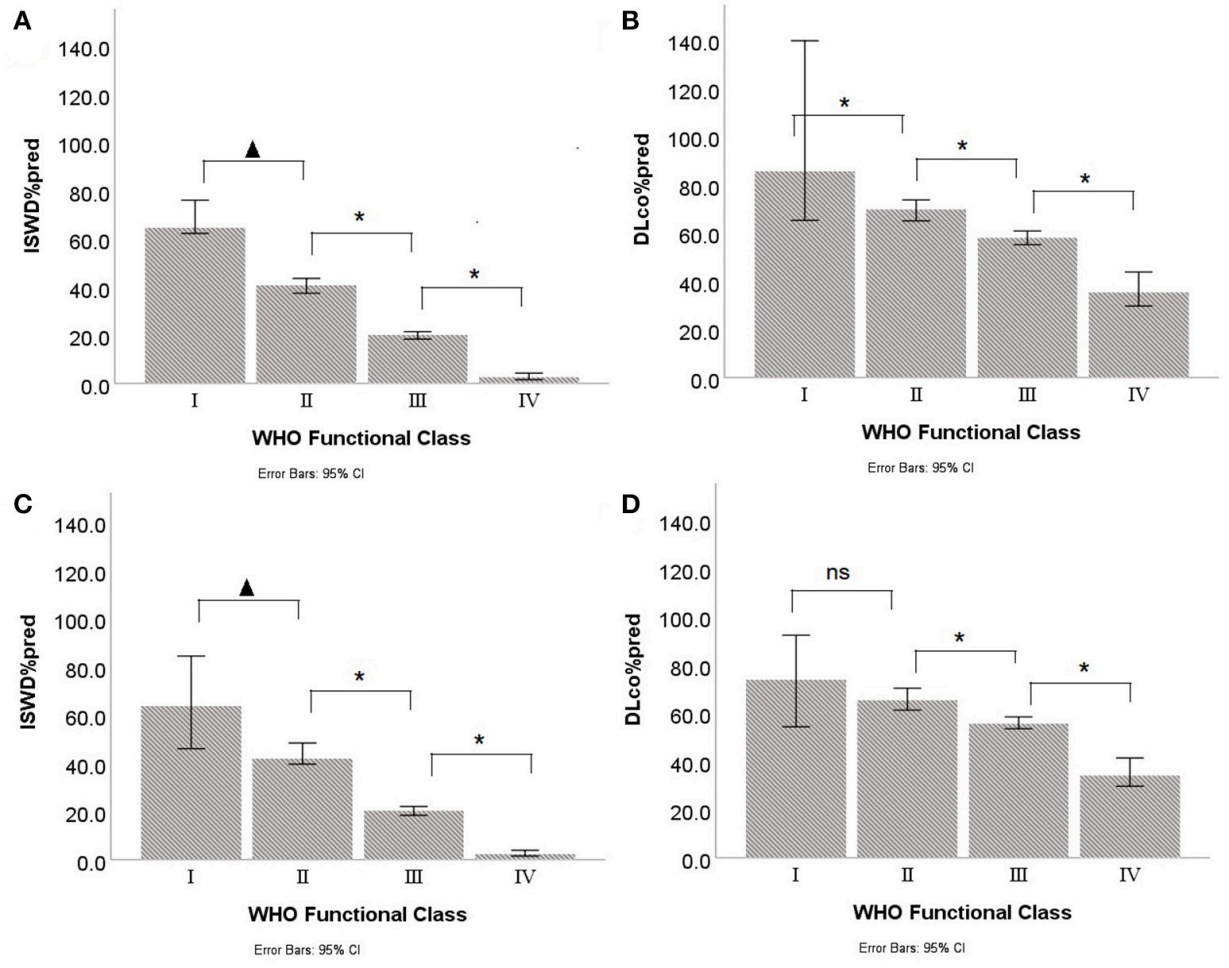
Median and interquartile ranges for Incremental shuttle walk distance percent predicted (ISWD%pred) and Diffusing capacity across the lung for carbon monoxide percent predicted (DLco%pred) by World Health Organization Functional Class (WHO FC) in **(A,B)** all patients and **(C,D)** with congenital heart disease patients excluded. *p* < 0.05; ^*^p < 0.005; ns, not significant.

Although there were significant correlations between WHO FC and ISWD%pred and DLco%pred, there was heterogeneity in ISWD%pred and particularly DLco%pred within each WHO FC (Figure 2). Figures 3A,B show the median and interquartile range of results for ISWD%pred and DLco%pred, respectively, for all patients by WHO FC. A large interquartile range was noted for DLco%pred in WHO FC I. When patients with congenital heart disease were omitted from analysis (Figures 3C,D) the interquartile range of DLco%pred in WHO FC I was reduced. However, although ISWD%pred still discriminated between patients in WHO FC I and II (63 ± 15 vs. 45 ± 23 *p* = 0.02), DLco%pred (74 ± 14 vs. 69 ± 17) and FVC%pred (96 ± 12 vs. 89 ± 23) were no longer significantly different (*p* > 0.05). Similar patterns of heterogeneity was seen in the frequency of standardized scores.

**Figure 4:**
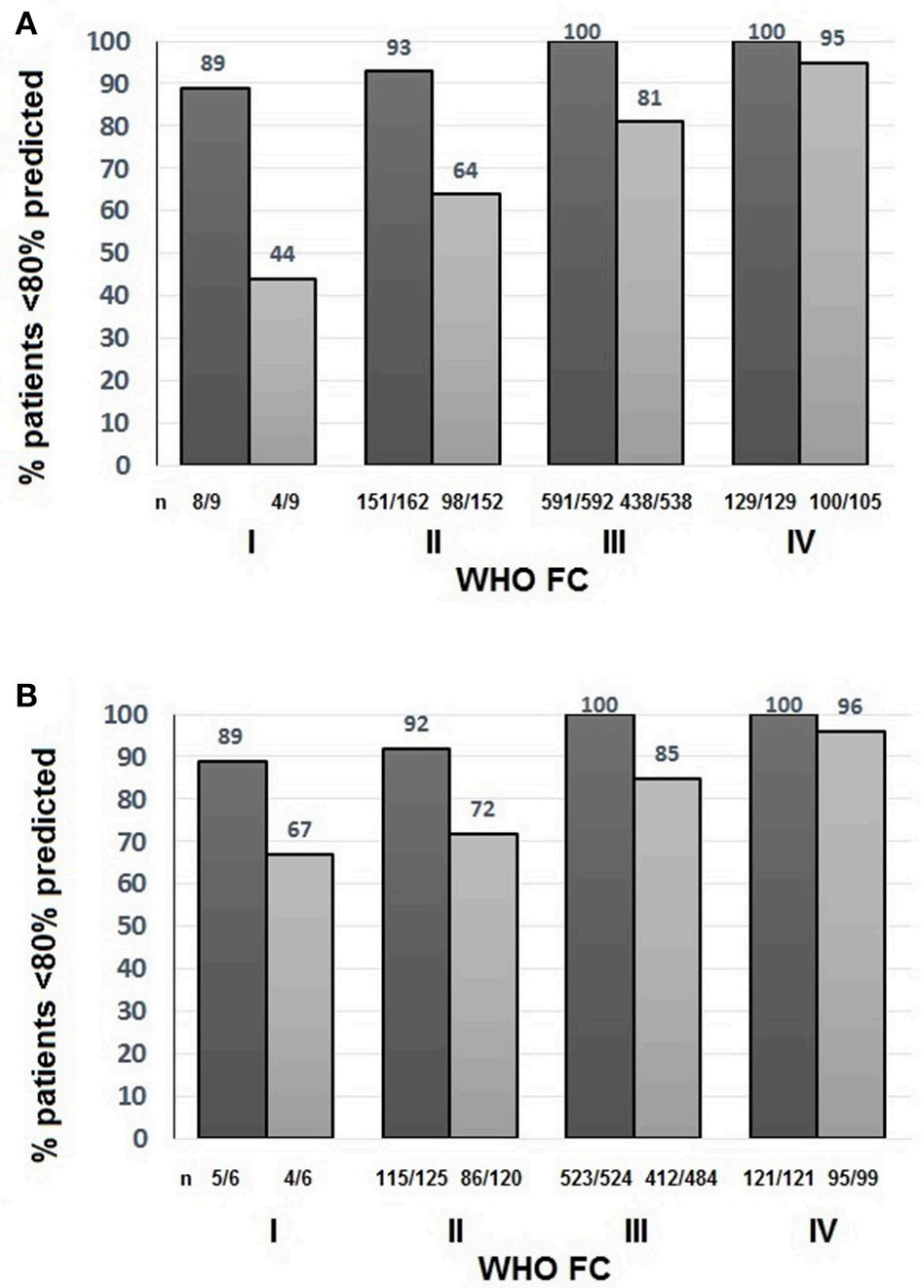
Percentage of patients with Incremental shuttle walk distance percent predicted (ISWD%pred) 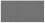 and Diffusing capacity across the lung for carbon monoxide percent predicted (DLco%pred) 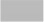 less than 80% predicted in **(A)** All patients **(B)** Patients with congenital heart disease excluded by World Health Organization Functional Class (WHO FC).

**Figure 5:**
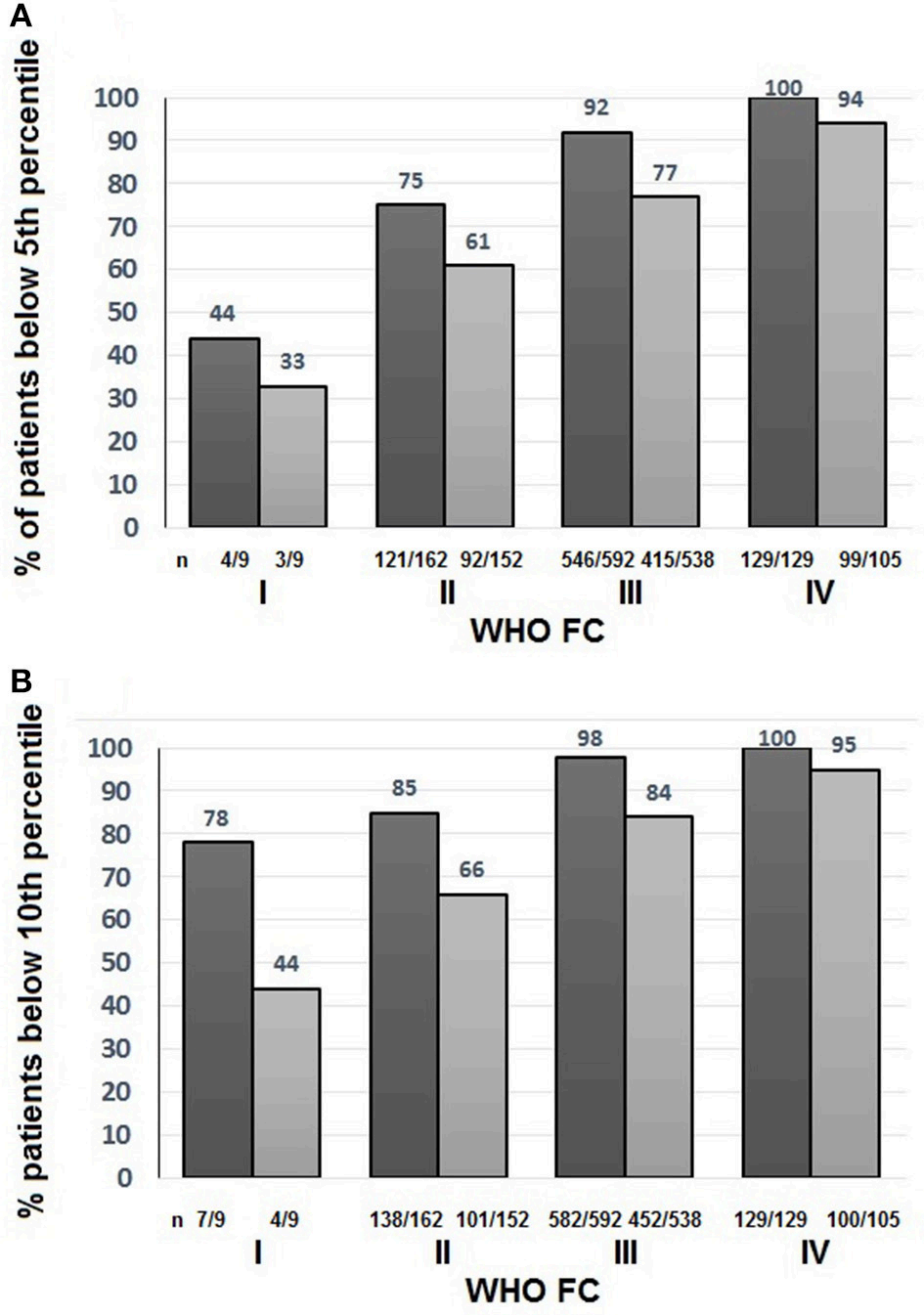
Percentage of all patients with Incremental shuttle walk distance percent predicted (ISWD%pred) 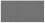 and Diffusing capacity across the lung for carbon monoxide percent predicted (DLco%pred) 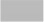 less than **(A)** the 5th percentile and **(B)** the 10th percentile World Health Organization Functional Class (WHO FC).

To assess the suitability of the ISWT as a screening tool, cut- off points using %pred and z-score were investigated. Figure 4 shows the percentage of patients with an ISWD%pred of <80%. A greater percentage of patients were identified using a cut-off of <80 ISWD%pred than by using the DLco%pred <80 cut-off. Figure 2 shows that for any given %pred cut-off point, ISWD%pred will positively identify more of the PH patients than DLco%pred. Using z scores, ISWD is again a better discriminator than DLco (Figure 5). In WHO FC 1 44% of patients had an ISWD below the 5th percentile and 78% were below the 10th percentile. Overall 73% of patients in WHO FC I and II are below the 5th percentile for ISWD compared to only 58% below the 5th percentile for DLco. Eighty four percent vs. 66% respectively were below the 10th percentile.

### Survival analysis

**Figure 6:**
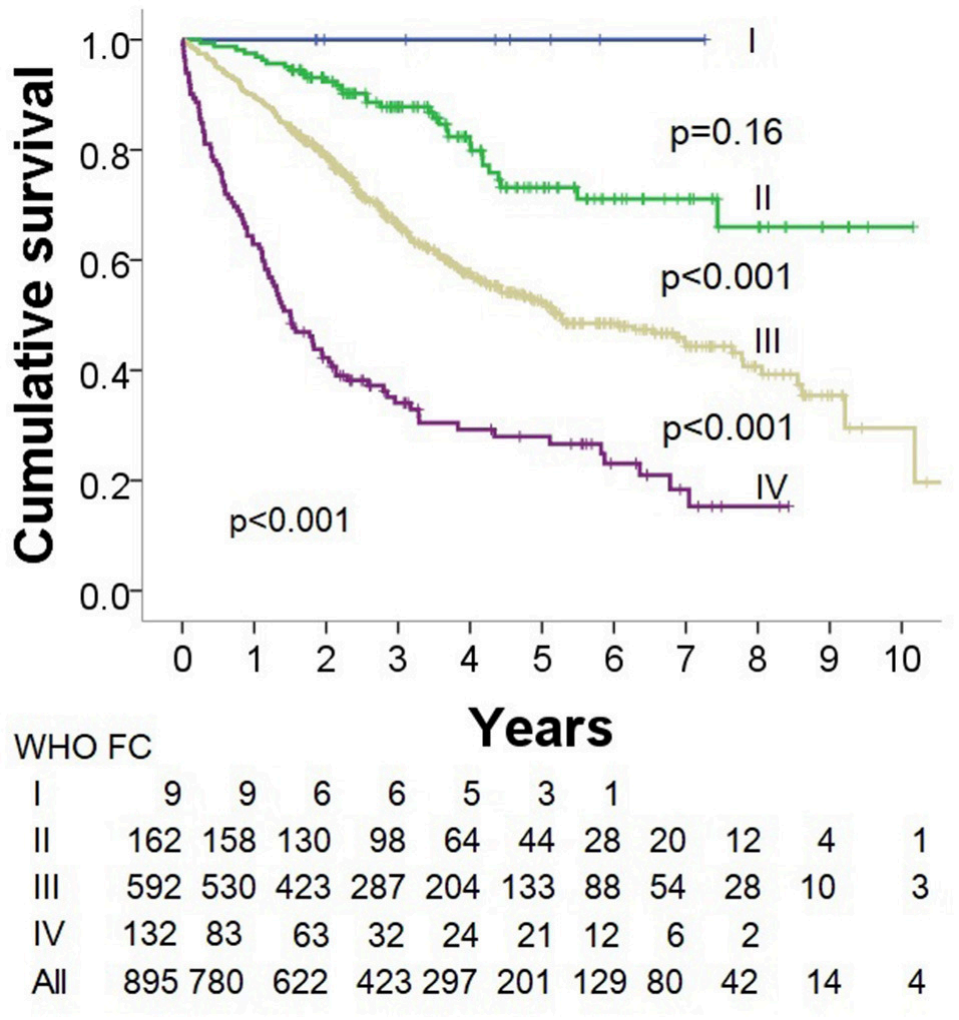
Kaplan Meier survival analysis by World Health Organisation functional class.

Kaplan-Meier survival analysis demonstrated that WHO FC was a significant predictor of outcome with decreasing survival with increasing WHO FC (*p* < 0.0001) (Figure 6). Multivariate Cox survival analysis including the parameters ISWD%pred, age, sex, BMI, mPAP and DLco showed that ISWD%pred remained a significant predictor (*p* < 0.001) (Table 3).

**Table 3:** Multivariate Cox survival analysis.

	**HR**	**95% CI**	***p***
ISWD%pred	0.983	0.975 – 0.990	<0.0001
Age	1.017	1.007 – 1.026	<0.0001
BMI	0.980	0.961 – 0.999	0.044
mPAP	1.022	1.011 – 1.032	<0.0001
DLco%pred	0.974	0.967 – 0.980	<0.0001

*ISWD%pred, Incremental shuttle walk test percent predicted; BMI, body mass index; mPAP, mean pulmonary arterial pressure; DLco%pred, diffusion factor across the lung for carbon monoxide percent predicted; HR, hazard ratio; CI, confidence interval*.

## Discussion

To our knowledge we have shown for the first time that patients with pulmonary hypertension in WHO FC I have a significant reduction in exercise capacity compared to predicted values. We have also demonstrated that exercise capacity is more sensitive than measurements of gas transfer made at rest in identifying patients with pulmonary hypertension in WHO FC I and WHO FC II. A single cut-off point is often suggested for screening as it is easy to remember and apply. We evaluated the use of a cut-off point of 80%pred looking for early changes and found that 89% of patients in WHO FCI were below this cut-off point. Whilst traditionally the normal range for a lung function parameters was considered as being between 80 and 120%pred it is known now that this could lead to misdiagnosis (28). We therefore also examined percentile scores as cut-off points and again found that the ISWT was a sensitive test with 73% of patients in WHO FC I and II below the 5th percentile for ISWD.

In addition to confirming the findings of previous studies (4, 29, 30) that WHO FC has a significant impact on survival, we have also demonstrated that in the absence of symptoms of breathlessness or in the presence of mild symptoms (i.e. patients in WHO FC 1/II) patients have a modest elevation of pulmonary artery pressure at initial diagnosis. In contrast patients with more severe symptoms of breathlessness (WHO FC III and IV) had significantly higher mean pulmonary artery pressure elevation. Therefore strategies to diagnose patients earlier when they have less symptomatic limitation is likely to identify patient with less severe pulmonary haemodynamic disease. Patients in WHO FC I included 3 patients with systemic sclerosis who had been identified from screening regimens and these patients had only mild elevation of mean pulmonary artery pressure, median 27 mmHg. Patients not undergoing regular screening, may also have been referred on the basis of echocardiograms performed for the assessment of incidental murmurs or on the basis of morphological changes consistent with pulmonary hypertension seen on cross sectional imaging.

It could be possible that there is some misclassification of the patients as classification into WHO FC is limited by patient and physician subjectivity and agreement between observers is often poor (31). There are however significant differences in ISWD%pred and DLco%predicted between FC I and II and there is a trend for increased survival. Breathlessness post ISWT is no greater in WHO FC I suggesting that the increased distance walked is not due to increased effort. This study, therefore, does emphasize that if we are to rely on self-reported symptoms of breathlessness to diagnose pulmonary hypertension then patients will have established hemodynamic changes of pulmonary hypertension at the time of diagnosis.

Current ESC/ERS guidelines recommend the use of Doppler echocardiography for screening for pulmonary hypertension in at risk patients. Like all screening tools echocardiography has limitations. In 10–20% of patients it is not possible to obtain interpretable results and the precision of echocardiography estimation of systolic pulmonary artery pressure can be poor (32). To overcome these difficulties other data, using lung function tests and utilizing gas transfer (which is reduced as a consequence of vascular involvement), have been used inscreening algorithms. Most work has been done in systemic sclerosis using DLco%pred. Hanchulla et al. (9) in a French multi-center trial found that a low DLco of <60% predicted was associated with a higher probability of PAH with only 30% of the newly diagnosed patients having a DLco > 60%pred. A UK study among 243 systemic sclerosis-associated PH patients found that <10% had a DLco >60%pred (10). Guidelines now suggest using a cut-off of DLco%pred <60% as part of the screening algorithm indicative of possible pulmonary hypertension in systemic sclerosis (33). This strategy has been shown to successfully enrich this population of patients undergoing right heart catheter to investigate possible pulmonary hypertension (11). However, whilst this algorithm has been used with some success in systemic sclerosis it is not applicable in patients with pulmonary hypertension associated with other aetiologies (34). Our study highlights the large range of DLco%pred found in WHO FC I, even when omitting patients with congenital heart disease. In contrast ISWD%pred correlated well with WHO FC with <10% of patients in WHO FC I and II having an ISWD >80%pred.

A number of studies have looked at the use of exercise testing including cardiopulmonary exercise testing, exercise Doppler echocardiography or diffusion capacity during exercise (35–39) to try to detect loss of compliance in the cardiopulmonary circulation earlier than parameters measured at rest but the tests used are complex. The advantage of the ISWT for screening is that it is a very simple test to perform and has been shown to reflect disease severity without a ceiling effect (8). Data from this study suggest it might be suited to detecting early disease. Pulmonary arterial hypertension and chronic thromboembolic pulmonary hypertension, for which specific therapies exist, are rare and therefore early identification relies on having a high degree of awareness in patients at risk and deploying appropriate disease specific strategies. Reducing the time to diagnosis and institution of treatment for patients with pulmonary hypertension requires a number of complimentary approaches and screening for pulmonary hypertension in at risk groups is only one approach. In addition to considering pulmonary hypertension in high risk groups (e.g., systemic sclerosis, portal hypertension, HIV, family history of PAH), there needs to be an increased awareness amongst patients to seek advice when they have exercise limitation and for physicians to more systematically assess the breathless patient. Our results suggest that exercise limitation (identified using a maximal exercise test) is an almost universal finding in patients with pulmonary hypertension even in patients who are asymptomatic. Further study is required to assess whether the incremental shuttle walking test could be used as a first line investigation or as part of a battery of tests to screen at risk patients, proceeding to more complex testing if abnormal.

## Limitations

This was a retrospective, single center study. Within the studied population only 1% of patients were diagnosed in WHO FC I and 18% in WHO FC II. These percentages are small (but are very similar to those found in the Geissen Pulmonary Hypertension Registry, which has 1.0% patients in WHO FC I, and confirm the findings in other registries (4, 26) that the majority of patients are diagnosed with PH in WHO FC III when symptomatic and haemodynamic severity are advanced. Given the small number of patients in WHO FC I, interpretation of negative results must be viewed with caution Classification of WHO FC may be limited by patient and physician subjectivity and agreement between observers is often poor (31). However in this study there appears to be a trend to increased survival in WHO FC 1 patients and ISWD is significantly higher in patients in WHO FC I. Although patient motivation could affect distance walked, the ISWT is externally paced which limits the effect of motivation and patients in WHO FC I did not report a higher dyspnoea score post-test suggesting that the difference in distance walked was not due to greater patient effort.

## Conclusion

Our results demonstrate that patients with newly diagnosed pulmonary hypertension with no or minimal symptomatic limitation have a significant reduction of exercise capacity.

## Author contributions

CB, IA, RC, and DK: study design; JH, RL, CB, IAS, MA, AC, CE, RC, and DK: performance of the research; CB, AT, CE, IS, AC, AL, and DK: data analysis; CB and DK: writing of the paper; All authors: revision of manuscript.

### Conflict of interest statement

The authors declare that the research was conducted in the absence of any commercial or financial relationships that could be construed as a potential conflict of interest.
